# Ionic Solvent Shell Drives Electroactuation in Organic Mixed Ionic‐Electronic Conductors

**DOI:** 10.1002/advs.202308746

**Published:** 2024-03-01

**Authors:** Filippo Bonafè, Francesco Decataldo, Tobias Cramer, Beatrice Fraboni

**Affiliations:** ^1^ Department of Physics and Astronomy University of Bologna Viale Berti Pichat 6/2 Bologna 40127 Italy

**Keywords:** electrochemical actuation, electrochemical atomic force microscopy, electroswelling, ion transport, organic mixed ionic‐electronic conductors

## Abstract

The conversion of electrochemical processes into mechanical deformation in organic mixed ionic‐electronic conductors (OMIECs) enables artificial muscle‐like actuators but is also critical for degradation processes affecting OMIEC‐based devices. To provide a microscopic understanding of electroactuation, the modulated electrochemical atomic force microscopy (mEC‐AFM) is introduced here as a novel in‐operando characterization method for electroactive materials. The technique enables multidimensional spectroscopic investigations of local electroactuation and charge uptake giving access to the electroactuation transfer function. For poly(3,4‐ethylenedioxythiophene) polystyrene sulfonate (PEDOT:PSS) based microelectrodes, the spectroscopic measurements are combined with multichannel mEC‐AFM imaging, providing maps of local electroactuation amplitude and phase as well as surface morphology. The results demonstrate that the amplitude and timescales of electroactuation are governed by the drift motion of hydrated ions. Accordingly, slower water diffusion processes are not limiting, and the results illustrate how OMIEC microactuators can operate at sub‐millisecond timescales.

## Graphical abstract

In this work, the modulated electrochemical atomic force microscopy (mEC‐AFM) is introduced to study microscopic electroactuation processes in organic mixed ionic‐electronic conductors. By combining spectroscopic measurements with multichannel mEC‐AFM imaging, it is demonstrated that electroswelling is governed by the drift motion of hydrated ions, achieving sub‐millisecond operation in PEDOT:PSS microactuators.

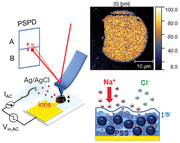

## 1 Introduction

Electroactive materials convert electrical energy to mechanical deformation,^[^ ^1^ ^]^ and have received considerable attention in the last years due to their envisioned and actual applications.^[^ ^2^ ^]^ An important example are organic materials with mixed ionic and electronic conductivity (OMIECs), that show the electrically‐induced swelling (electroswelling) causing actuation based on contraction or expansion of the overall volume.^[^ ^3^ ^]^ Such process can be favorably exploited in devices able to mimic the functionalities of biological muscles (artificial muscles), with the OMIEC specific advantages of biocompatibility, low‐voltage drive, nanoscale precision, miniaturization, and operation in liquid environments.^[^ ^4^ ^]^ At the same time, OMIECs find application in energy storage,^[^ ^5^ ^]^ bioelectronic transducers^[^ ^6^ ^]^ and organic electrochemical transistors,^[^ ^7^ ^]^ where volume changes are potentially causing device failure as the continuous expansion and contraction of the electroactive material causes interfacial strain leading ultimately to delamination.^[^ ^8^ ^]^ Despite the relevance of electroactuation in OMIECs in different fields of material research, fundamental knowledge on the intrinsic mechanism of electroswelling is still lacking. A deeper comprehension of the process could explain a major issue of OMIEC‐based actuators, i.e., their low operation speed. OMIECs produce at least ten times more force for a given cross‐sectional area than skeletal muscle,^[^ ^9^ ^]^ but their operation is reported to be 100 times slower.^[^ ^10^ ^]^ During volume change, OMIECs translate an external stimulus to a change of the physical properties on a nanometer scale,^[^ ^2^ ^]^ but the dynamics of the process are not completely clear. Filling this knowledge gap could establish quantitative metrics for both the timescale and width of electroswelling, guiding the development of enhanced material formulations with larger electroactivity or stability according to the application.

Electroswelling in OMIECs is driven by the oxidation or reduction of the electronic conductor and the associated charge compensation in the ionically conducting phase to maintain overall charge neutrality.^[^ ^11^ ^]^ The consequent exchange of cations or anions and solvent between the OMIEC and the electrolyte bath causes the swelling/deswelling of the film.^[^ ^12^ ^]^ Electrochemical stimuli can be applied locally and in a controlled way, and as a result, ionic electroactive thin films show a strong potential for applications.^[^ ^13^, ^14^ ^]^ Starting from the pioneering work of Baughman,^[^ ^15^ ^]^ and Pei and Inganas,^[^ ^16^ ^]^ different OMIECs soft‐actuators were developed and applications ranging from microfluidic valves,^[^ ^17^ ^]^ drug delivery devices,^[^ ^18^ ^]^ and sensing microstructures to microgrippers,^[^ ^19^ ^]^ refreshable displays,^[^ ^18^ ^]^ and microrobots^[^ ^20^ ^]^ were demonstrated. Among OMIECs, material selection is mainly focused on polypyrrole (PPy), which has been fully studied and can be used in large‐scale production.^[^ ^21^ ^]^ PPy thin films generate large actuation strain and stress,^[^ ^22^ ^]^ but also suffer from several drawbacks, such as high rigidity, low conductivity, ion diffusion rate, and the risk of over‐oxidation.^[^ ^23^ ^]^ A significant alternative to PPy is Poly(3,4‐ethylenedioxythiophene) (PEDOT) doped with polystyrene sulfonate (PEDOT:PSS).^[^ ^1^ ^]^ In PEDOT:PSS, electronic transport is achieved by mobile hole charges present in the oxidized semiconducting polymer PEDOT. The positive charge of the holes is counterbalanced by immobile, negative ionic charges of the polyanion PSS.^[^ ^24^ ^]^ Oxidized organic semiconductor and ionic polyanion form a nanophase separated network that generates close electrostatic interaction between the two phases. Consequently, high electronic carrier mobility (*µ* > 10 cm^2^ V^−1^ s^−1^)^[^ ^25^ ^]^ is combined with a strong volumetric capacitive coupling (*c_v_* > 30 F cm^−3^).^[^ ^26^ ^]^ PEDOT:PSS conductivity is one order of magnitude higher than that of PPy and can be further improved by blending or post‐treatment.^[^ ^27^ ^]^ The highly stable oxidized state enables to maintain conductivity for several months, considerably enhancing the cycling stability of PEDOT:PSS‐based actuators,^[^ ^23^ ^]^ and making PEDOT:PSS a model material for quantitative measurements of electroactuation.

Electroswelling in OMIECs has been investigated using quartz crystal microbalance measurements with dissipation monitoring (EC‐QCM‐D) during cyclic voltammetry (CV).^[^ ^12^ ^]^ Through EC‐QMC‐D it is possible to monitor mass exchange between an electrically active film and an electrolyte as CV induces electrochemical doping/de‐doping of the film.^[^ ^28^ ^]^ EC‐QMC‐D was successfully used to study the electrochemical actuation of ferrocyanide‐containing polyelectrolyte multilayers,^[^ ^12^ ^]^ but also the ionic‐to‐electronic coupling efficiency in PEDOT:PSS,^[^ ^28^ ^]^ or the charge carrier dynamics in organic electrochemical transistors.^[^ ^29^ ^]^ Other relevant characterization methods involve UV–vis absorption to study equilibrium concentration of polaronic and neutral species at different swelling conditions,^[^ ^30^ ^]^ in situ X‐ray diffraction to reveal the microstructure variation during electrochemical processes,^[^ ^31^ ^]^ or in situ X‐ray scattering,^[^ ^32^ ^]^ and NMR^[^ ^33^ ^]^ to probe the changes in ion coordination.^[^ ^34^, ^35^ ^]^ At the same time, molecular dynamics simulations focusing on understanding water intake and ion exchange in PEDOT:PSS were developed to interpret experimental results.^[^ ^36^ ^]^ Also electrochemical atomic force microscopy (EC‐AFM)^[^ ^37^, ^38^ ^]^ was used to follow changes in thickness in Ppy^[^ ^39^ ^]^ and PEDOT:PSS^[^ ^40^ ^]^ films, probing possible morphological changes induced by swelling. However, these methods all required macroscopic electroactive thin films and could only reveal slow actuation processes occurring on the timescale of seconds and the monitoring of fast local ionic exchange processes remains elusive. As a faster and more local technique, electrochemical strain microscopy was introduced to study ion‐exchange processes in P3HT, but as only the tip generates the local electric field, results are of qualitative nature.^[^ ^41^ ^]^

In this work, we address this issue introducing the modulated electrochemical force microscopy (mEC‐AFM) as novel operando characterization method for electrochemical actuation, combining both microscopy and spectroscopy of electroswelling. We exploit AFM as a local probe for volume changes and interface forces that provides transient data on dynamical effects related to electroactuation in OMIECs. Combination of the electroswelling data with impedance spectroscopy in PEDOT:PSS microelectrodes yields a multidimensional spectroscopy revealing the dominant timescales for ion migration and electroswelling. The resulting knowledge allows us to implement multichannel mEC‐AFM imaging, providing maps of local electroswelling amplitude and phase as well as surface morphology. The results demonstrate that the amplitude and timescales of electroswelling are governed by the drift motion of hydrated ions. Accordingly slower water diffusion processes are not limiting and microactuators can operate at frequencies exceeding several kHz.

## 2 Results

### 2.1 mEC‐AFM Spectroscopy of Electroswelling in PEDOT:PSS

**Figure 1:**
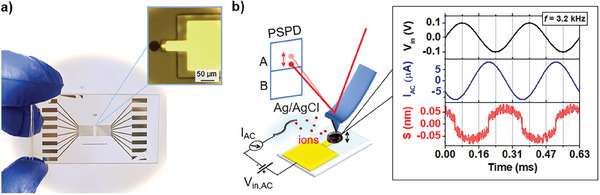
Experimental setup. a) Optical image of a PEDOT:PSS microelectrode array used for the AFM experiment. The structure of a single electrode is provided by the magnified image shown as an inset. b) Schematic of the experimental setup for mEC‐AFM, showing the AFM operating in contact mode on a microelectrode when an AC voltage is applied. The signals measured during the experiment are plotted as a function of time. The frequency of the input voltage *V_in_* is *f =* 3.2 kHz.

**Figure** **1**a shows an optical image of the investigated micro‐structured PEDOT:PSS electrodes. Two rows of 8 gold microelectrodes are symmetrically patterned onto a glass substrate, allowing for each electrode the electrodeposition of a PEDOT:PSS layer with controlled thickness. Multiple electrodes are designed on the same substrate to vary systematically the thickness of the electroactive polymer layer. Also, a higher magnification image of a single electrode with 50 µm diameter is shown featuring a PEDOT:PSS layer of 1 µm thickness. A thick negative photoresist layer (3 µm) electrically insulates the gold feedline from the surrounding electrolyte. Figure 1b shows a schematic of the experimental setup used for the modulated electrochemical atomic force microscopy (mEC‐AFM) measurements. In this technique, we use a frequency modulation to induce electrochemical doping/de‐doping of an OMIEC material and we acquire the local thickness variations induced by the process. Samples are immersed in a 0.1 m PBS solution, using a Ag/AgCl wire as reference electrode, and the AFM probe is brought into contact with the surface of the PEDOT:PSS layer (force set point 10 nN, using NSC36 probes of the Park NX10 AFM).

The application of a sinusoidal modulation *V_in_* (with amplitude |*V_in_* | = 100 mV and angular frequency *ω*) to the microelectrode drives the reversible oxidation and reduction processes of the PEDOT:PSS layer. Concurrently, the exchange of ions between the electrolyte and the PEDOT:PSS film assures charge neutrality and gives rise to the AC current signal.^[^ ^42^ ^]^ The continuous injection and extraction of ions into and out of the PEDOT:PSS leads to the swelling and deswelling of the soft polymeric matrix at the same frequency as the drive signal. The resulting thickness oscillation is measured by acquiring the vertical deflection of the AFM cantilever in contact with the film surface. Fast adjustments of tip height are avoided as the oscillation frequencies are kept above the bandwidth of the z‐scanner feedback‐loop control (see Section S1, Supporting Information). Typical data traces obtained by this procedure are shown in Figure 1b as a function of time: they include the input voltage applied by the AC voltage source, the resulting AC microelectrode current and the AFM height changes. All signals are observed here at the fundamental frequency of *f* = 3.2 kHz as provided by the AC signal source.

**Figure 2:**
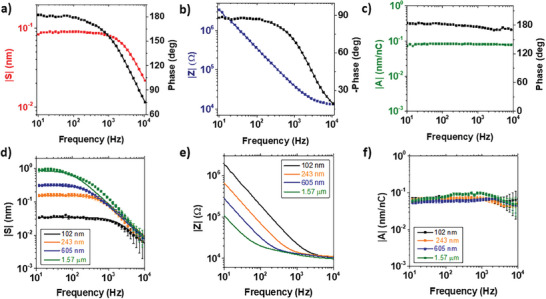
Multidimensional electroswelling spectroscopy. a) Electroswelling spectrum obtained by sweeping the frequency of the AC input voltage in a wide range. Spectra of the b) electrochemical impedance and c) actuation transfer functions relating charge uptake and swelling in the polymer matrix. d) Electroswelling, e) impedance, and f) actuation function amplitudes measured on PEDOT:PSS layers with different thicknesses. The electrode diameter is 50 µm. Error bars indicate the average between measurements performed in five different positions on the electrode surface. Experimental data in (d) (indicated with squares) are fitted assuming a constant value *a* for the actuation function for each thickness. Fitting results are reported in Table 1.

**Figure 3:**
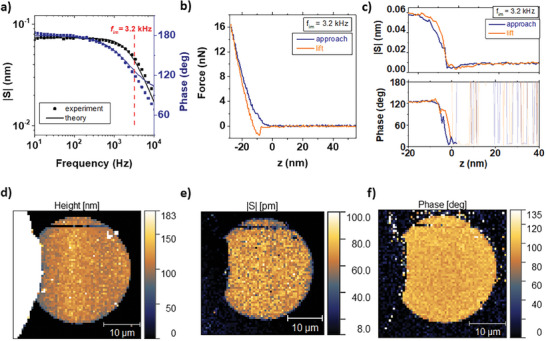
mEC‐AFM imaging of electroswelling amplitude and phase on a PEDOT:PSS covered microelectrode. a) Electroswelling spectrum of the 30‐µm electrode, the frequency *f_im_* applied to the input voltage *V_in_* during the image acquisition is indicated. b,c) Simultaneous measurement of force‐distance spectroscopy (b) and electroswelling amplitude and phase (c) on a single pixel. The repetition of this process allows for the imaging of the electrode height (d), e) electroswelling amplitude, and f) phase on the entire electrode region.

**Figure 4:**
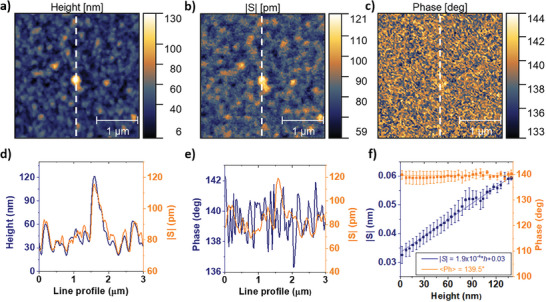
Nanoscale mEC‐AFM imaging of the electroswelling effect. Multichannel AFM acquisition of a) the topography, b) electroswelling amplitude, and c) phase on a PEDOT:PSS microelectrode. The imaging frequency *f_im_* was set to 3.2 kHz. d,e) Line profiles extracted on the vertical axis of the images comparing the acquired data. f) Correlation between the local thickness, electroswelling amplitude and phase. Error bars are obtained dividing the height distribution in 32 bins and calculating the standard deviation of each histogram bar.

To perform spectroscopic measurements, a multichannel lock‐in amplifier is introduced to demodulate the AC signals, measuring the voltage, current and swelling phasors $\bar{V}\left(\omega \right)$, $\bar{I}\left(\omega \right)$ and $\bar{S}\left(\omega \right)$. The last is obtained by measuring the phasor corresponding to the vertical deflection of the AFM cantilever and dividing its amplitude by the cantilever sensitivity (see Experimental Section and Section S1, Supporting Information). The resulting spectrum (**Figure** **2**a) shows how the swelling amplitude has a plateau in the low frequency regime with a stable phase of 180°. In this condition, the local height of the PEDOT:PSS layer increases by |*S*| when the AC voltage reaches its minimum value (−100 mV). Then, after a critical frequency, the phase shift reduces, and the swelling amplitude decreases with increasing frequency. This behavior can be better understood considering the transfer functions relating to the three measured phasors. The electrochemical impedance transfer function $Z\;=\bar{V}\;/\bar{I}$ reveals how the electrolyte limits charge entering the polymer film. The resulting Bode plot (Figure 2b) shows how the impedance amplitude is limited by the electrolyte resistance at high frequency but increases in the low frequency range where the swelling plateau is measured. In such a regime, the phase of the corresponding current reaches 90°, indicating capacitive charge accumulation in the PEDOT:PSS layer. The second relevant transfer function is the actuation function $A\;=\;\bar{S}/\bar{Q}$, where $\bar{Q}=\;\int_{0}^{t}\bar{I}\left(s\right)ds$ is the phasor defining the charge accumulated in the polymer film (see Section S3, Supporting Information). The actuation function *A* reveals how the morphology of the soft polymer matrix changes through the injection of charge. The spectrum of *A* is reported in Figure 2c and illustrates how its amplitude remains constant across all frequencies, with a phase of 180°. We remark that the electroswelling measurements are local, while AC current measurements involve the entire electrode area. To assure reproducibility we performed electroswelling spectroscopies on 5 different points on the polymer surface and find experimental variations in the transfer functions that are <5% (Figure 2a,c, see also **Figures** **3** and **4** for the mapping of the amplitude and phase of *S*). In the spectroscopy experiments we also varied systematically the drive amplitude up to *V_in_* = 200 mV and found a linear response of the current and swelling signal (Section S4, Supporting Information). This finding is in agreement with the absence of faradaic reactions in PEDOT:PSS over a broad electrochemical potential window.^[^ ^25^ ^]^

From this evidence, we can formulate an interpretation for electroswelling in PEDOT:PSS. When a positive voltage is applied to the microelectrode, the PEDOT phase gets oxidized and positive hole charges enter the film. To maintain charge neutrality, the ionic PSS phase is depleted from cations, leading to a decrease in the film volume. Instead upon reduction (negative voltage) hole carriers are removed from the PEDOT phase and cations must enter the film to counterbalance the negative charge present in the PSS phase. As a consequence, the film increases in local height by |*S*|, when a negative voltage is applied, explaining the antiparallel response of *A* (180°) with respect to the voltage signal.

Our observations are confirmed performing experiments on electrodes with different PEDOT:PSS thicknesses (Figure 2d–f). Analysis of the two transfer functions reveals that electroswelling is only limited by the impedance of the PEDOT:PSS/electrolyte interface. Consequently, the amplitude of *A* (Figure 2f) has a constant value *a* independently on both, the film thickness as well as frequency. Following this observation, we can model the impedance spectra in Figure 2e with an equivalent RC circuit to calculate the charge accumulated in the film, and then fit the swelling spectra keeping the “actuation coefficient” *a*, as the only adjustable parameter (see Section S5, Supporting Information). Results (continuous lines in Figure 2d) show an excellent agreement with the experimental points. The related fit parameters are reported in **Table** **1** and show values for *a* compatible within the experimental errors. Two important conclusions arise from the multidimensional spectroscopy: i) fast electroswelling can be obtained if sufficient charge can be injected. The timescale of actuation is provided by the characteristic time of the RC circuit *τ = RC*. ii) The actuation function has a constant value *a* for the PEDOT:PSS formulation independent on both the frequency and the amount of injected charge. This identifies *a* as an intrinsic metric of the actuation extent and allows for a microscopic interpretation which will be discussed in the final paragraph.

**Table 1:** Quantitative analysis of impedance and electroswelling spectroscopies on PEDOT:PSS electrodes with diameter of 50 µm, resulting from the fits in Figure 2d,e.

Thickness	R [kΩ]	C [*nF*]	[nm nC^−1^]
102 ± 6 nm	10.4 ± 0.8	9.2 ± 0.8	0.063 ± 0.008
243 ± 8 nm	11.4 ± 1.2	24 ± 2	0.076 ± 0.002
605 ± 10 nm	11.3 ± 1.4	57 ± 3	0.080 ± 0.006
1.6 ± 0.5 µm	10.1 ± 1.8	147 ± 8	0.075 ± 0.007

### 2.2 mEC‐AFM Imaging of Electroswelling in PEDOT:PSS

Next, we aim to investigate the impact of the local morphology on electroswelling properties. Similar to piezoelectric force microscopy, mEC‐AFM maps transient electroactuation on the polymer surface by acquiring the amplitude and phase of the surface height oscillations induced by an AC voltage modulation. Such a mEC‐AFM imaging experiment can be implemented on microelectrode surfaces with sufficiently small RC constant to allow for electroswelling at frequencies extending into the kHz range. Achieving fast electroswelling operation is essential to perform stable mEC‐AFM image acquisitions with a reasonable scan time. Figure 3a shows the electroswelling spectrum for a 30‐µm diameter electrode. For imaging experiments on this electrode, we used a modulation frequency of *f_im_* = 3.2 kHz that is in the frequency roll‐off region but still offers a good signal to noise ratio, indicating that a significant amount of charge is capacitively accumulated in the bulk of the PEDOT:PSS layer and contributes to the swelling process. Image acquisitions performed at different frequencies are reported in Section S6 (Supporting Information). For the soft polymer surface, we use fast approach and retract curves to avoid shear forces and potential tip or sample damage during scanning. As shown in Figure 3b, for each pixel of the image, the tip is approached until a threshold force of *F_max_* = 16.7 nN is reached. During the same approach, the surface oscillation amplitude and phase are recorded (Figure 3c). Both signals increase only after the AFM tip gets into contact with the PEDOT:PSS layer (indicated with *z =* 0). Upon increased force, the swelling amplitude and phase become stable and remain constant, indicating that the threshold force *F_max_* applied on the sample during the spectroscopy does not hinder the oscillation of the electroactive polymer surface. It also allows us to exclude other distant dependent forces such as electrostatic interactions between tip and sample as origin of the observed oscillations. Once above threshold force, the amplitude and phase signals are averaged for *t_hold_* = 1 ms. Then the AFM probe is lifted from the contact position, and the operation is repeated for the following pixel. Images obtained by the mEC‐AFM technique on the entire microelectrode region (50 × 50 µm) are shown in Figure 3d–f and represent the surface height, the swelling amplitude and the swelling phase. A constant electroswelling and stable phase are only measured on the PEDOT:PSS covered microelectrode. The encapsulation layer and the substrate show changes in surface morphology, but no electroswelling signal (Figure 3e,f).

Next, we acquire a multichannel mEC‐AFM image with increased resolution imposing 128 × 128 pixels on a region of 3 × 3 µm in the center of the microelectrode (Figure 4). The height (Figure 4a) and electroswelling amplitude (Figure 4b) show a strong correspondence in the image. The combined line profiles in Figure 4d demonstrate that increased electroswelling is measured in regions of the film surface where the PEDOT:PSS layer is thicker. Instead, the signal phase (Figure 4c) is not affected by the local morphology and maintains a constant value. The quantitative analysis of the correlations is depicted in Figure 4f that relies on a statistical analysis of all pixels of the image. A linear correspondence between the local PEDOT:PSS film height and the local electroswelling amplitude is observed. The finding is rationalized by considering the intrinsic volumetric capacitance of PEDOT:PSS. By considering *c_v_* = 28 ± 2 Fcm^−3^,^[^ ^43^ ^]^ and the electrode area *A_el_*, we can calculate the actuation coefficient from the slope *k* of the correlation line as $a\;=\frac{k}{A_{el}\left|V_{in}\right|c_{v}}\;$ = 0.09±0.01 nm nC^−1^, obtaining results consistent with the spectroscopy experiments shown in Figure 2. This quantitative agreement explains nicely the contrast observed in Figure 3b and provides additional evidence that the local electroswelling is directly correlated to the number of ions entering the film below the AFM tip.

## 3 Discussion

Our experimental observations demonstrate that charge accumulation is intrinsically related to volume change in PEDOT:PSS. Two different hypotheses can be initially formulated to account for the dynamics of the swelling behavior. The first hypothesis (that we can define “energetic”) predicts that hydrated ions are driven with their water shell inside PEDOT:PSS by the external electrical potential *V_in_*. The second hypothesis (that we can define “entropic”) assumes that “bare” ions are injected into the electroactive material and water uptake is caused by a subsequent osmotic process. Despite the possibility to model the PEDOT:PSS layer as a semi‐permeable membrane due to the presence of the fixed PSS^−^ acceptors,^[^ ^44^ ^]^ our spectroscopy measurements highlight how osmosis plays only a minor role in the electroswelling mechanism. In the entropic interpretation, the actuation timescale would be limited by the diffusive transport of water at the polymer/electrolyte interface. Modeling experiments of water uptake in PEDOT:PSS allow for the quantification of the water diffusion coefficient in the material as *D_w_* ≈ 10^−12^ m^2^s^−1^.^[^ ^45^ ^]^ Using this value and assuming the complete hydration of the polymeric layer on the vertical axis, we can estimate the diffusion time as $t_{d}=\frac{t^{2}}{4D_{w}}\;$, where *t* is the thickness of the PEDOT:PSS film. Typical timescales for the 300 nm thick films of 22.5 ms (see Supp. Inf. 7) clearly illustrate that water diffusion is a relatively “slow” process with respect to electroswelling and validate the “energetic” interpretation. In this view, we can directly associate the actuation coefficient *a* to the dimension of the hydrated radius in the PEDOT:PSS layer. If we assume that each injected ion is responsible for the electronic doping/de‐doping of the material, we can calculate the volume of hydrated ions as *v_ion_* = A_el_*e*a (see Supp. Inf. 8), and correlate the hydration radius *r_ion_* to the actuation coefficient:

(1) $$r_{ion}={\left(\frac{3aA_{el}e}{4\pi }\right)}^{1/3}$$

where *e* is the elementary charge. By replacing the average actuation coefficient *<a>* = 0.075±0.007 nm nC^−1^ from Table 1, we obtain *<r_ion_>* = 0.18±0.02 nm.

**Figure 5:**
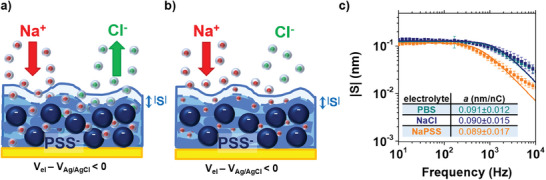
Interpretation of electroswelling in PEDOT:PSS. a) Ion transport involves both cations and anions inside their water shell, with a smaller fraction of anions leaving the material when a negative potential is applied to the electrode. b) Ion transport involves only cations, and their water shell gets smaller inside the material. c) Electroswelling spectroscopies performed in different electrolytes. Results are a strong support for the hypothesis in (b). Error bars indicate the average between measurements performed in 5 different positions on the electrode surface.

The measured radius of hydration *<r_ion_>* in PEDOT:PSS corresponds to half the value typically measured for Na^+^ cations in water (*r_Na,w_* = 0.36 nm).^[^ ^46^ ^]^ The reduction in hydration radius can be interpreted by alternative descriptions: i) when cations enter inside the polymeric matrix, a considerable fraction of anions is ejected, reducing the swelling extent (**Figure** **5**a), or ii) only cations are responsible for the electroswelling, but their water shell inside PEDOT:PSS gets smaller (Figure 5b). A final, third hypothesis is provided by a combination of the two. To properly identify that one of the three scenarios best fits with the ion dynamic inside the material, we performed electroswelling spectroscopies in three different electrolytes (0.1 m PBS, 0.14 m NaCl and 0.4 mm NaPSS). These differ from pH (being 7.38 ± 0.02, 6.24 ± 0.02, and 3.94 ± 0.02 for the 0.1 m PBS, 0.14 M NaCl and 0.4 mM NaPSS, respectively), sodium cation concentration, and anion composition. Results are reported in Figure 5c and demonstrate equal actuation coefficients for the three different electrolytes. As a consequence, measurements exclude a significant role for the PSS protonation/deprotonation in the active swelling, despite its major importance in the passive water uptake causing material hydration.^[^ ^36^ ^]^ The findings in NaPSS solution, allow us to exclude the transport of anions as PSS^−^ are too large to enter the polymer films. Still, the swelling coefficient does not differ from the one measured in PBS and NaCl solutions, proving that chloride anions do not substantially contribute to the electroswelling, and ion uptake is limited only to sodium cations. This observation strongly supports the swelling mechanism illustrated in Figure 5b. Fixed PSS^−^ acceptors limit the uptake of mobile anions from the surrounding electrolyte by electrostatic repulsion, while coordination to sulfate groups causes smaller hydrodynamic radius *<r_ion_>* = 0.18 ± 0.02 nm for cations. Similar results are expected to be obtained in solutions containing (mixtures of) different monovalent cations, as their free water hydration radii do not substantially differ.^[^ ^46^ ^]^ The experimental *<r_ion_>* value is consistent with previous analyses performed using an electrochemical quartz crystal microbalance (EQCM), estimating an uptake of 1–2 water molecules for every injected ion.^[^ ^28^ ^]^

The results obtained in this work demonstrate how modulated electrochemical force microscopy (mEC‐AFM) can be successfully applied to reveal the intrinsic dynamics of electrochemical actuation in OMIEC materials. We prove that the amplitude and timescales of electroswelling are governed by the drift motion of hydrated ions and not limited by slower water diffusion processes. Accordingly, we demonstrate how fast ionic charging of the PEDOT:PSS volumetric capacitance leads to high frequency operation in microactuators, and how the swelling extent is intrinsically limited by the size of the ionic solvent shell. The resulting knowledge can serve as a guideline for the development of OMIEC‐based actuators with maximized performances. The work also shows how mEC‐AFM offers novel means to interrogate materials with mixed ionic‐electronic conductivity at the nanoscale during operation providing information on the local ionic distribution and the ion‐to‐electron coupling. This capability will be critical to understand more complex OMIEC‐based realizations such as organic electronic ion pumps^[^ ^47^ ^]^ or organic electrochemical transistors.^[^ ^48^ ^]^

## 4 Experimental Section

### Device Fabrication

Glass substrates (50 × 25 mm^2^) were cleaned by sonication in acetone/isopropanol/distilled water baths. After a dehydration step (10 min at 110° C), the Microposit S1818 positive photoresist was spin coated (4000 rpm for 60 s) and annealed at 110 °C for 1 min. Metallic contacts were patterned through direct writing lithography by using the ML3 Microwriter (from Durham Magneto Optics). The photoresist was developed with Microposit MF‐319 developer. Then, 7 nm of chromium and 30 nm of gold were deposited by thermal evaporation. Samples were immersed in acetone for 4 h for photoresist lift‐off. Metallic contacts were encapsulated with the mr‐DWL 5 negative photoresist (from Micro Resist Technology). The resin was spin coated at 3000 rpm for 30 s and annealed at 100 °C for 2 min. After laser exposure, samples were baked at 100 °C for 2 min and relaxed for 1 h at room temperature. Development was performed with mr‐Dev 600 developer (Micro Resist Technology), and the resist was finally hard‐baked at 120 °C for 30 min. PEDOT:PSS was electropolymerized on the electrode surface starting from an aqueous solution containing 3,4‐Ethylenedioxythiophene (EDOT) (10 mm) and Poly(sodium 4‐styrenesulfonate) (NaPSS) (0.1 mm) (both from Merck). Polymeric layers of controlled thicknesses were obtained through a two‐electrodes galvanostatic procedure (current density 2 mA cm^−2^), using the Keysight 2912A source‐measure unit (SMU) and a Ag/AgCl wire as counter/reference electrode.

### mEC‐AFM Spectroscopy of Electroswelling

Atomic force microscopy (Park System's NX10 AFM) was performed in liquid, using 0.1 m PBS as electrolyte and an Ag/AgCl wire as reference electrode. The cantilever sensitivity *s_c_* of NSC36 probes was measured through force‐distance spectroscopies on the glass substrate of the samples. The probe was lifted to the vertical coordinate *z_0_* = 0.1 µm from the contact position, then gradually approached to the sample surface (scan speed 0.3 µm s^−1^) down to *z_1_* = −0.2 µm and finally retracted again to *z_0_*. *s_c_* was extracted from the slope of the linear repulsive interaction measured in contact regime (see Section S2, Supporting Information). AC measurements were performed with the MFLI lock‐in amplifier (from Zurich Instruments). A constant zero DC offset voltage and a sinusoidal oscillation (amplitude 100 mV) with desired frequency were applied to the PEDOT:PSS electrode with respect to the Ag/AgCl reference electrode. The AFM tip was pushed in contact mode on the sample surface (force set point 10 nN), and the gain of the z scanner was set to 0.01 to slow down the feedback response allowing the acquisition of the surface vibration from the cantilever deflection signal (see Section S1, Supporting Information). Different NSC36 probes were used during the experiment to ensure the independence of the acquired deflection on the tip radius. The AC current flowing in the PEDOT:PSS layer and the deflection signal were demodulated to measure the impedance and electroswelling spectra of the samples. A complete scheme of the experimental setup is reported in Section S9 (Supporting Information). During each acquisition, the frequency of the sinusoidal modulation was swept between 10 and 10^4 ^Hz. Electroswelling measurements were repeated on five different points for every PEDOT:PSS electrode coating.

### mEC‐AFM Imaging of Electroswelling

Multichannel images were obtained on 30 µm diameter electrodes by operating the Park NX10 AFM in PinPoint mode. This allows to simultaneously acquire the topography and a high‐speed force‐distance curve for every pixel of the scan area. The basic operation parameters were set as follows: cantilever approach/retract speed = 20 µm s^−1^, force set point = 20 nN, z‐servo duration = 4 ms, average time = 1 ms, pixel‐to‐pixel move time = 3 ms. In parallel, we measured the amplitude and phase of the electroswelling signal with the lock‐in amplifier and sent them as input to the AFM software to determine their mean value during the average time. The frequency of the input voltage *V_in_* was kept constant to 3.2 kHz. Applying such fast modulation, the amplitude and phase of the surface height oscillation due to electroswelling can be scanned for a whole surface area in reasonable time. Multichannel images were acquired both on an entire electrode region (64 × 64 pixels on a 50 × 50 µm scan area) and on a specific area of the PEDOT:PSS layer (128 × 128 pixels on a 3 × 3 µm scan area). For the second case, cantilever retract speed was increased to 40 µm s^−1^ to decrease the acquisition time. The z‐scanner motion range was restricted to 3 µm.

## Conflict of Interest

The authors declare no conflict of interest.

## Supporting information

Supporting Information

## Data Availability

The data that support the findings of this study are available from the corresponding author upon reasonable request.
