# Supplementary material for: Ionic Solvent Shell Drives Electroactuation in Organic Mixed Ionic‐Electronic Conductors
Source: Adv Sci (Weinh). 2024 Mar 1;11(18):2308746. doi: 10.1002/advs.202308746 (PMC11095215; doi:10.1002/advs.202308746)
Supplement: Supplementary file 1 — Supporting Information [file ADVS-11-2308746-s001.pdf]

ADVANCED SCIENCE
Open Access

Supporting Information

for *Adv. Sci.*, DOI 10.1002/advs.202308746

Ionic Solvent Shell Drives Electroactuation in Organic Mixed Ionic-Electronic Conductors

Filippo Bonafè, Francesco Decataldo, Tobias Cramer and Beatrice Fraboni*

Supporting Information

1. AFM acquisition of the electroswelling-induced oscillations

During the acquisition of the electroswelling spectra, fast adjustments of the z-scanner height are avoided as the AC oscillation frequencies are kept above the bandwidth of the z-scanner feedback-loop control. When operating in contact mode, the AFM feedback-loop acts to counteract variations in the vertical deflection signal, consequently acquiring the topography of the sample. By changing the z-servo gain parameter, we can modify the bandwidth of the feedback-loop. Figure S1a shows the vertical deflection and the z-height signals measured during the acquisition of an electroswelling spectrum (NSC36 probe, force set point 10 nN). The gain is set to 0.1. From normalized data, we observe that the feedback loop starts to effectively follow the sample oscillation only below a certain frequency f_c. Consequently, the deflection signal is suppressed, and the height signal increases. The position of f_c can be tuned by modifying the gain parameter (Figure S1b and S1c). Setting the z servo gain to 0.01 in our system (Figure S1c) allows for a stable acquisition of the vertical deflection induced by electroswelling in the frequency range of our experiment.

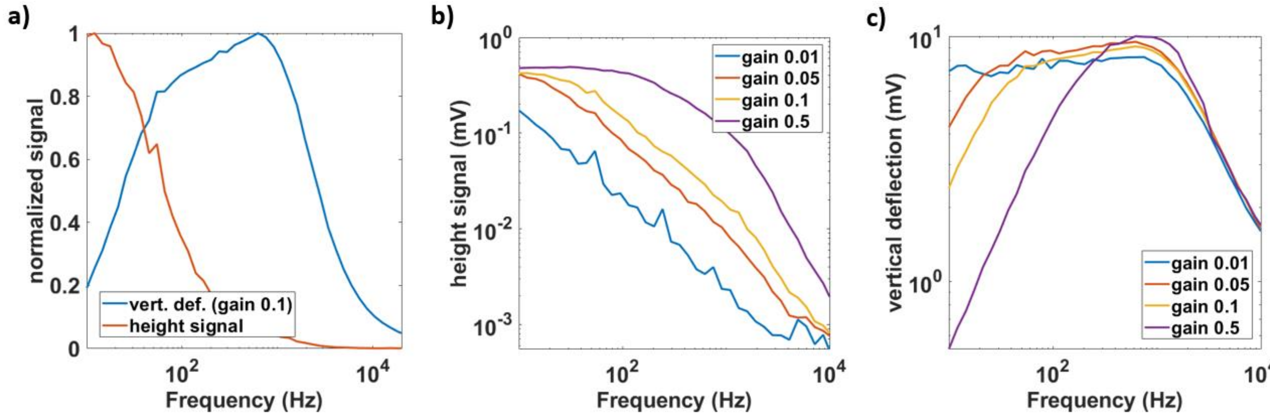

Figure S1: Adjustment of the AFM z-servo gain parameter to maximize the acquisition of the electroswelling-induced oscillations on the surface of the sample. a) Normalized z-height and vertical deflection signals as a function of the frequency of V_{in}. The gain is set to 0.1. b) Height and vertical deflection (c) signals measured varying the z-servo gain.

2. Measurement of the cantilever sensitivity and calculation of the swelling amplitude

We measured the cantilever sensitivity s_c of NSC36 probes in liquid through force-distance spectroscopies on the rigid glass substrate of the samples (Figure S2a). The probe was lifted to the vertical coordinate z_0 = 0.1 μm from the contact position, then gradually approached to the sample surface (scan speed 0.3 μm/s) down to z_1 = -0.2 μm and finally retracted again to z_0. The resulting force is calculated by dividing the acquired vertical deflection D (A-B voltage in the AFM position-sensitive photodiode (PSPD)) by the cantilever elastic constant (Figure S2b). $s_c = dD/dz$ was extracted from the slope of the linear cantilever deflection signal measured in contact regime (Figure S2c). During electroswelling measurements, the force set point for contact mode operation was set to 10 nN to have a significative interaction between the probe and the sample. After applying the V_{in} signal, we calculated the swelling amplitude as $|S| = |D|/s_c$, where $|D|$ is the amplitude of the vertical deflection of the AFM cantilever caused by the sample oscillation.

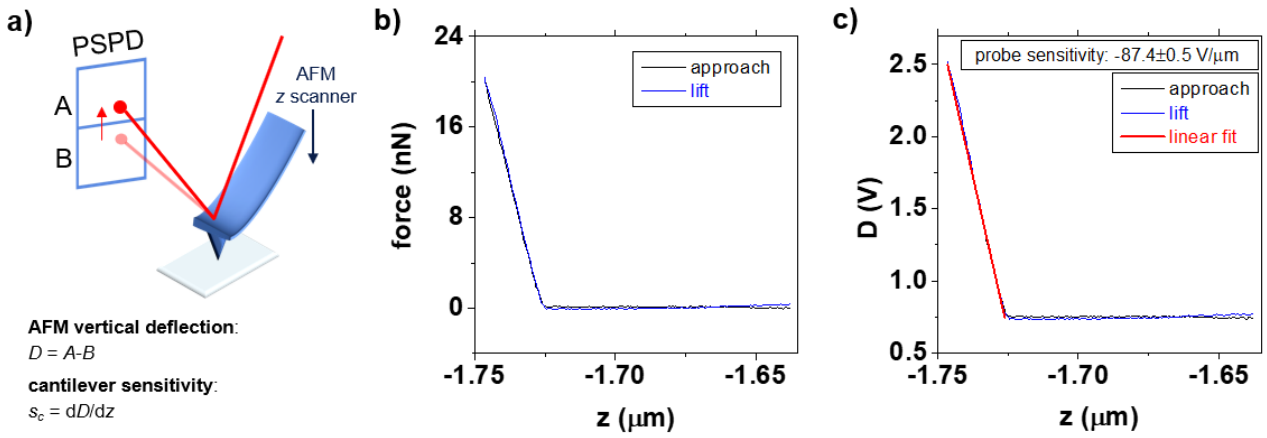

Figure S2: a) Experimental setup used to measure the NSC36 cantilever sensitivity. b) Force-distance spectroscopy on the glass substrates of the sample. c) Determination of the cantilever sensitivity.

3. Derivation of the charge phasor

In an AC circuit, we can define the charge phasor as the time integral of the current phasor:

$$\bar{Q} = \int \bar{I} dt \qquad (S3.1)$$

$$\bar{Q} = \int |I| e^{j(\omega t + \phi_I)} dt = \frac{|I| e^{j(\omega t + \phi_I)}}{j\omega} = \frac{\bar{I}}{j\omega} \qquad (S3.2)$$

thereby, we obtain

$$\bar{Q}(t) = \frac{|I|}{\omega} e^{j(\omega t + \phi_I - \frac{\pi}{2})} = |Q| e^{j(\omega t + \phi_Q)} \qquad (S3.3)$$

with

$$|Q| = \frac{|I|}{\omega}; \qquad \phi_Q = \phi_I - \frac{\pi}{2} \qquad (S3.4)$$

4. Linearity of the electrochemical response of PEDOT:PSS microactuators

We ensure the linearity of the mEC-AFM characterization method by measuring the electroswelling amplitude for different amplitudes of the applied AC voltage modulation. Experimental data reported in Supp. Inf. 4 confirm a linear electroactuation response from PEDOT:PSS, and the actuation coefficient resulting from the fitting line slope results to be compatible with experimental results reported in Table 1. This finding also furtherly confirms the frequency dependent model of the

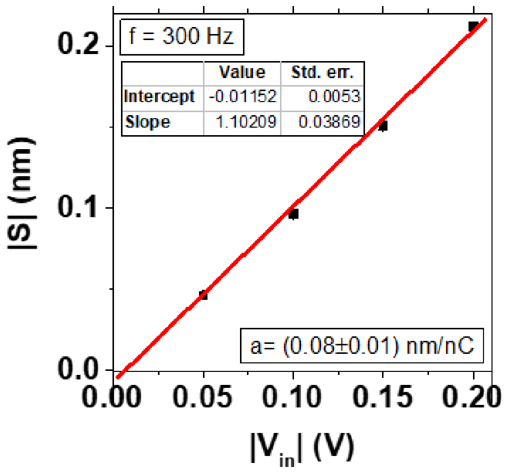

Figure S4: Linear electrochemical response of PEDOT:PSS microactuators. The actuation coefficient can be calculated from the slope of the fitting line using Eq. S5.6.

swelling phasor (Supp. Inf. 5). Eq. S5.6 predicts that when the frequency is fixed, the actuation amplitude can be linearly controlled through different amplitudes of the input voltage determining the amount of charge injected in the polymeric matrix.

5. Frequency dependence model of the swelling phasor

The PEDOT:PSS/electrolyte interface can be modeled with an equivalent RC series circuit, composed by the ionic resistance of the electrolyte R and the capacitance C of the electric double layer in the polymeric layer. The input voltage phasor $\overline{V_{in}}(t) = |V_{in}|e^{j\omega t}$and the current phasor $I(t) = |I|e^{j(\omega t+\phi_I)}$ are related through the electrochemical impedance transfer function:

$$Z = \frac{1}{j\omega C} + R \qquad \text{(S5.1)}$$

Using the impedance, we can express the charge phasor as a function of the input voltage phasor:

$$\bar{Q}(t) = \int \bar{I}dt = \int \frac{|V_{in}|}{\frac{1}{j\omega C} + R} e^{j\omega s}ds \qquad \text{(S5.2)}$$

we obtain

$$\bar{Q}(t) = \frac{C|V_{in}|}{\sqrt{1 + (\omega RC)^2}} e^{j[\omega t + \mathrm{atan}\,(-\omega RC)]} \qquad \text{(S5.3)}$$

with amplitude and phase

$$|\bar{Q}| = \frac{C|V_{in}|}{\sqrt{1 + (\omega RC)^2}}; \qquad \phi_Q = atan(-\omega RC) \qquad \text{(S5.4)}$$

We can finally relate charge and swelling phasors through the actuation function:

$$A = \frac{\bar{S}}{\bar{Q}} \qquad \text{(S5.5)}$$

The experimental spectroscopy data shows that A has a constant value $A(\omega) = a$ over the experimental frequency range. Accordingly, we can write the mathematical expressions for the swelling amplitude and phase:

$$|\bar{S}| = \frac{aC|V_{in}|}{\sqrt{1 + (\omega RC)^2}}; \qquad \phi_S = atan(-\omega RC) \qquad \text{(S5.6)}$$

obtaining a good agreement with experimental results in Figure 2d.

6. Selecting the imaging frequency for mEC-AFM microscopy experiments

In mEC-AFM measurements in Figure 3 and 4 we identified f_{im} = 3.2 kHz as a good compromise between fast electroswelling operation allowing for image acquisitions with a reasonable scan time and good signal to noise ratio. Experiments performed at different frequencies are reported in Figure S6, where we compare line scans acquired on the PEDOT:PSS film at three different frequencies. On one hand, shifting f_{im} towards smaller values (f_1 = 1.7 kHz) increases the electroswelling amplitude in Figure S6b (and, thereby, the signal-to-noise ratio), but a longer time interval (t_{hold} = 1.5 ms) is required to acquire the slower polymer surface oscillation measured for every pixel, increasing the image scan time by 50%. On the other hand, increasing f_{im} to f_3 = 5.1 kHz allows for faster acquisitions, but reduces the electroswelling extent and thus the signal-to-noise ratio of the final image. Noteworthy, the acquisition of the local film morphology is not affected by the selected imaging frequency, as demonstrated by Figure S6c.

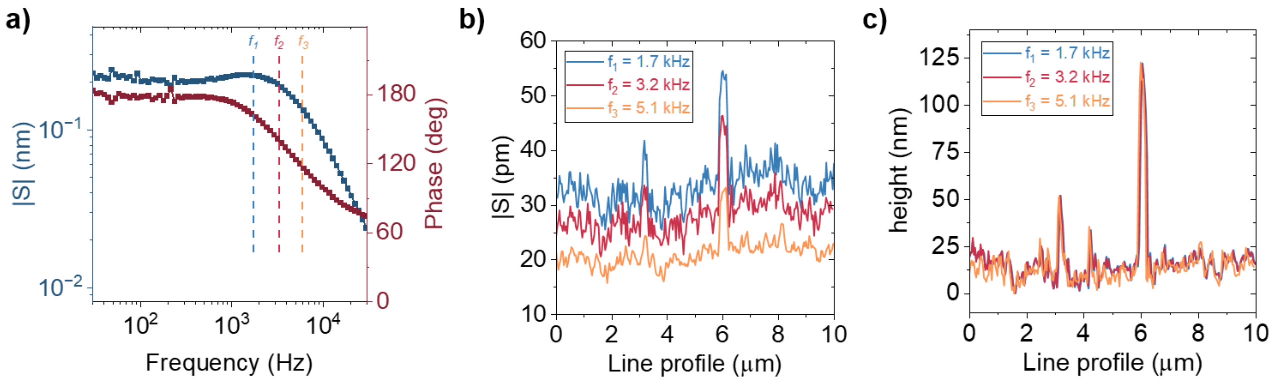

Figure S6: a) Electroswelling spectroscopy indicating 3 different imaging frequencies selected to acquire line scans on the PEDOT:PSS layer. f_2 corresponds to f_{im} in Figure 3 and 4. b) Swelling amplitude (b) and height (c) profiles measured along the same line at three different frequencies.

7. Water diffusion and osmosis in electroactuation of conductive polymers

Electroswelling spectroscopy measurements highlight how osmosis plays a minor role in the electroactuation mechanism. Using $D_w \approx 10^{-12}$ m^2/s as the water diffusion coefficient in PEDOT:PSS[3] and assuming the complete hydration of the polymeric layer on the vertical axis, we can estimate the diffusion time as $t_d = \frac{t^2}{4D_w}$, where t is the thickness of the PEDOT:PSS film. Results obtained for our experiment are reported in Table S7. These clearly illustrate that water diffusion is a relatively "slow" process with respect to electroswelling. Assuming osmosis as responsible for the latter implies a substantial discrepancy between the charge injected in the material and its volume change for frequencies higher than $f_d = 1/t_d$, which is not observed in the experiments (Figure 2b and 2c). Moreover, no significative increases in the actuation width are measured when $f < f_d$. The actuation timescale is not limited by the diffusive transport of water at the polymer/electrolyte interface caused by an increase of the osmotic pressure, but by the transport of hydrated cations.

**thickness (nm)**	**t_d (s)**	**f_d (Hz)**
102	0.002601	384.4675125
243	0.01476225	67.74035123
605	0.09150625	10.92821529
1570	0.616225	1.622783886

Table S7: Estimation of the timescale (t_d) and characteristic frequency (f_d) of water diffusion in PEDOT:PSS

8. Calculation of the ion hydration radius

If we assume that each injected ion is responsible for the electronic doping/de-doping of the material, we can express the swelling amplitude as a function of the number of ions as

$$S = aN_{ion}e \qquad \text{(S8.1)}$$

where a is the actuation coefficient (expressed in nm/nC), N_{ion} the total number of ions, and e the elementary charge. Spectroscopy data combined with the imaging experiment demonstrate that the charge uptake in the polymeric matrix is uniformly distributed within the film volume. Thereby, the volume change V_s induced by ionic injection is $V_s = A_{el}S = N_{ion}(aA_{el}e)$, where A_{el} is the electrode area. This identifies the volume of a single ion as

$$v_{ion} = aA_{el}e \qquad \text{(S8.2)}$$

and its radius will be

$$r_{ion} = \left(\frac{3v_{ion}}{4\pi}\right)^{1/3} = \left(\frac{3aA_{el}e}{4\pi}\right)^{1/3} \qquad \text{(S8.3)}$$

9. Detailed schematic of the experimental setup used for mEC-AFM experiments

A complete schematic of the experimental setup used for mEC-AFM experiments is reported in Figure S9a. A function generator connected to the PEDOT:PSS microelectrode provides the AC input voltage and DC offset (vs Ag/AgCl) leading to swelling/deswelling of the OMIEC layer. The same signal is given as a reference to the lock-in amplifier. Both the AFM cantilever deflection resulting from the PEDOT:PSS surface oscillations and the AC current flowing in the electrochemical cell are acquired by the lock-in amplifier. This demodulates the provided signals measuring the amplitude and phase of the voltage, current, and swelling phasors. The amplitude of the latter is calculated as $|S|= D/s_c$ according to Supp. Inf. 2. An optical image of the liquid cell containing both the samples and the AFM probe during mEC-AFM experiments is reported in Figure S9b. A polyether ether ketone (PEEK) substrate supports a PEDOT:PSS microelectrode array microfabricated on a glass slide. The PBS electrolyte is confined above the sample by a PEEK well sealed with screws and an underlying polydimethylsiloxane (PDMS) O-ring. A Ag/AgCl wire is placed on the right side of the well acting as reference electrode. The microstructured PEDOT:PSS structures acting as working electrodes are aligned with the center of the well and individually addressable though the metallic contact pads patterned at the left and right sides of the well.

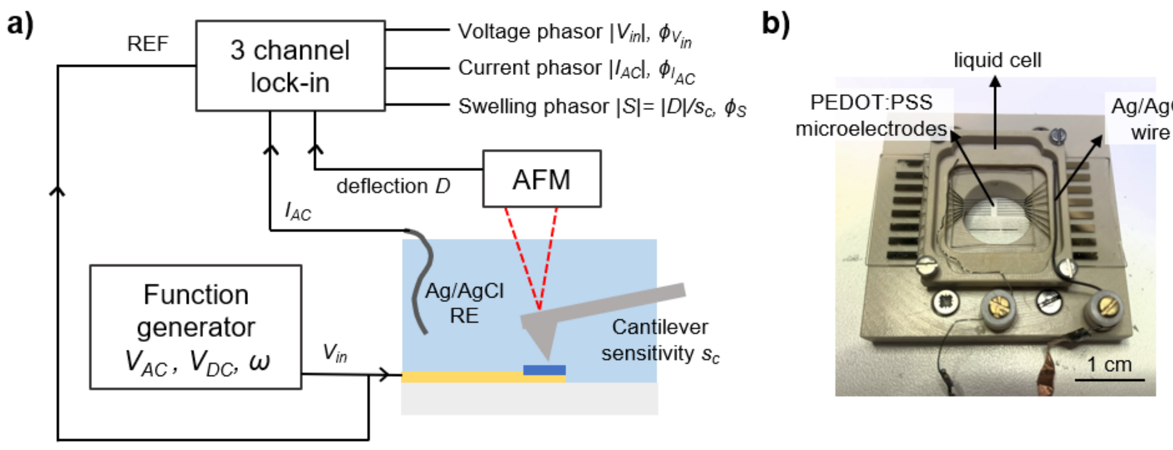

Figure S9: a) Detailed schematic of the experimental setup used for mEC-AFM experiments. b) Liquid cell used for in-liquid mEC-AFM experiments, featuring a Ag/AgCl wire as reference electrode on the right. Microstructured PEDOT:PSS electrodes are fabricated on a glass slide and aligned with the center of the cell. Microelectrodes are individually addressable through the contact pads placed at the left and right sides of the liquid cell. The setup also includes a platinum wire (left side of the cell) to enable the potentiostatic control of the system.
